# Supplementary material for: Early Anticoagulation in Patients with Acute Ischemic Stroke Due to Atrial Fibrillation: A Systematic Review and Meta-Analysis
Source: J Clin Med. 2022 Aug 25;11(17):4981. doi: 10.3390/jcm11174981 (PMC9457225; doi:10.3390/jcm11174981)
Supplement: Supplementary file 1 [file jcm-11-04981-s001.zip › jcm-1863050-supplementary/Supplementary Files.pdf]

ONLINE SUPPLEMENT

Complete search algorithm used in MEDLINE search.

Complete search algorithm used in SCOPUS search.

Supplementary Tables: S1, S2, S3, S4

Supplementary Figures: S1-S49

References used in the Supplement.

Complete search algorithm used in MEDLINE search.

("ischemic stroke"[All Fields] OR "stroke"[All Fields]) AND ("atrial fibrillation"[All Fields]) AND ("anticoagulation"[All Fields] OR "anticoagulant"[All Fields] OR "vitamin K antagonist"[All Fields] OR "warfarin"[All Fields] OR "NOAC"[All Fields]) OR "DOAC"[All Fields] OR "direct oral anticoagulant"[All Fields]) AND ("early"[All Fields]) AND ("initiation"[All Fields])

Complete search algorithm used in SCOPUS search.

(TITLE-ABS-KEY (({ischemic stroke} OR)) AND TITLE-ABS-KEY ({atrial fibrillation}) AND TITLE-ABS-KEY (({anticoagulation} OR { anticoagulant} OR { vitamin K antagonist} OR { warfarin} OR {NOAC} OR {DOAC} OR {direct oral anticoagulant})) AND TITLE-ABS-KEY ({early}) AND TITLE-ABS-KEY ({initiation}))

Supplementary Table-S1. Table of excluded studies with reasons for exclusion.

Study Name	Reason(s) for exclusion
Altavilla et al.[1]	No early initiation of anticoagulation - Bridging with low molecular weight heparin
Butcher et al.[2]	No history of atrial fibrillation
Cappellari et al.[3]	Overlapping data
De Marchis et al.[4]	Overlapping data
Paciaroni et al.[5]	Overlapping data
Seiffge et al.[6]	Overlapping data
Toyoda et al.[7]	Overlapping data
Tsivgoulis et al.[8]	Overlapping data
Wilson et al.[9]	Overlapping data
Yaghi et al.[10]	Retrospective design
Yaghi et al.[11]	Bridging with low molecular weight heparin

Supplementary Table-S2. Quality assessment of included cohort studies using the Risk Of Bias In Non-randomized Studies of Interventions (ROBINS-I) tool.[12]

Study name	Confounding	Selection	Classification of Interventions	Deviation from Interventions	Missing Data	Measurements of Outcomes	Reporting	Overall
Al Bakr et al. [13]	Serious	Low	Low	Low	Low	Low	Low	Serious
Alrohimi et al. [14]	Not Applicable	Low	Not Applicable	Not Applicable	Low	Low	Low	Low
Alrohimi et al. [15]	Not Applicable	Low	Not Applicable	Not Applicable	Low	Low	Low	Low
Gioia et al. [16]	Not Applicable	Low	Not Applicable	Not Applicable	Serious	Low	Low	Serious
SATES [17]	Not Applicable	Low	Not Applicable	Not Applicable	Serious	Low	Low	Serious
Seiffge et al. [18]	Serious	Low	Low	Low	Low	Low	Low	Serious

Supplementary Table-S3. Overview of the baseline characteristics of the patients included in the single-arm analysis according to the different time windows of initiating oral anticoagulants.

Variable	Time Windows	Prevalence			Test for Subgroup Differences
		N of studies	Pooled estimates (95%CI)	I^2, p for Cochran Q	
Women	Within 1 week	4	48.4% (36.8-60.2%)	91%, <0.001	p= 0.68
	Within 2 weeks	6	47.8% (37.3-59.0%)	92%, <0.001	
	Overall	8	48.3% (37.3-59.2%)	98%, <0.001	
Age (years)	Within 1 week	4	73 (68-77.9)	98%, <0.001	p= 1
	Within 2 weeks	6	73.2 (70.5-76)	90%, <0.001	
	Overall	8	73.3 (71.7-75)	96%, <0.001	
NIHSS	Within 1 week	4	5 (2-8)	99%, <0.001	p= 0.76
	Within 2 weeks	6	6 (2-9)	99%, <0.001	
	Overall	8	5 (3-7)	99%, <0.001	
CHA_2DS_2-VASc	Within 1 week	4	4 (3-5)	99%, <0.001	p= 0.65
	Within 2 weeks	4	4 (4-5)	98%, <0.001	
	Overall	6	4 (4-5)	99%, <0.001	
HAS-BLED	Within 1 week	3	2 (1-4)	99%, <0.001	p= 0.66
	Within 2 weeks	3	3 (2-4)	98%, <0.001	
	Overall	4	3 (2-3)	99%, <0.001	

Lesion Volume (ml)	Within 1 week	1	5 (4-7)	NA	p= 0.83
	Within 2 weeks	3	5 (2-8)	89%, <0.001	
	Overall	4	5 (3-7)	87%, <0.001	
Prior Stroke	Within 1 week	3	14.5% (4.8-28.1%)	91%, <0.001	p= 0.74
	Within 2 weeks	3	16.1% (14.5-17.8%)	0%, 0.390	
	Overall	4	16.6% (11.9-21.9%)	91%, <0.001	
Hypertension	Within 1 week	4	73.1% (66-79.6%)	77%, 0.005	p= 0.31
	Within 2 weeks	3	82.3% (62.6-95.6%)	95%, <0.001	
	Overall	5	76.6% (71.7-81.2%)	89%, <0.001	
Dyslipidemia	Within 1 week	3	25.1% (14.9-36.9%)	92%, <0.001	p= 0.13
	Within 2 weeks	2	47.5% (15.2-81.0%)	98%, <0.001	
	Overall	4	32.8% (26.1-39.9%)	94%, <0.001	
Diabetes Mellitus	Within 1 week	4	23.3% (18.4-28.6%)	63%, 0.046	p= 0.14
	Within 2 weeks	3	36.7% (18.5-57%)	94%, <0.001	
	Overall	5	26.8% (22.1-31.7%)	87%, <0.001	
Chronic Kidney Failure	Within 1 week	2	19.9% (18.5-21.3%)	0%, 0.978	p= 0.82
	Within 2 weeks	2	22.9% (8.7-41.3%)	85%, 0.009	
	Overall	2	19.3% (15.6-23.3%)	83%, 0.001	

Supplementary Table-S4. Overview of the baseline characteristics of the patients included in the pairwise analysis comparing DOACs versus VKAs, stratified by the treatment initiation.

Variable	Time Windows	Effect			Test for Subgroup Differences
		**N of studies**	**Estimates (95%CI)**	**I^2, p for Cochran Q**	
Women	Within 1 week	2	RR 0.78 (0.49-1.26)	86%, 0.007	p= 0.008
	Within 2 weeks	2	RR 1.57 (1.29-1.92)	38%, 0.20	
	Overall	3	RR 1.08 (0.58-2.04)	99%, <0.001	
Age (years)	Within 1 week	2	MD -0.03 (-0.83 – 0.78)	0%, 0.80	p= 0.96
	Within 2 weeks	2	MD -0.06 (-1.12 – 0.99)	0%, 0.55	
	Overall	3	MD -0.04 (-0.68 – 0.60)	0%, 0.94	
NIHSS	Within 1 week	2	MD -0.34 (-3.67 – 2.99)	97%, <0.001	p= 0.35
	Within 2 weeks	2	MD -1.95 (-2.61- -1.29)	0%, 0.46	
	Overall	3	MD -0.92 (-2.41 – 0.56)	91%, <0.001	
CHA_2DS_2-VASc	Within 1 week	2	MD 0.14 (-0.23 – 0.52)	62%, 0.11	p= 0.32
	Within 2 weeks	2	MD -0.15 (-0.61 – 0.30)	50%, 0.16	
	Overall	3	MD 0.02 (-0.18 – 0.22)	43%, 0.15	
HAS-BLED	Within 1 week	2	MD 0.06 (-0.12 – 0.24)	60%, 0.11	p= 0.50
	Within 2 weeks	1	MD -1.00 (-1.04 - -0.96)	NA	
	Overall	2	MD -0.27 (-1.07 – 0.52)	100%, <0.001	

	Within 1 week	1	RR 0.75 (0.63-0.89)	NA	p< 0.001
Prior Stroke	Within 2 weeks	2	RR 1.51 (1.18-1.92)	0%, 0.46	
	Overall	2	RR 1.06 (0.58-1.94)	91%, <0.001	
	Within 1 week	2	RR 1.03 (0.89-1.18)	49%, 0.16	
Hypertension	Within 2 weeks	2	RR 1.01 (0.90-1.14)	78%, 0.03	p= 0.87
	Overall	3	RR 1.01 (0.95-1.08)	65%, 0.04	
	Within 1 week	2	RR 0.81 (0.74-0.90)	0%, 0.96	
Dyslipidemia	Within 2 weeks	2	RR 0.69 (0.54-0.87)	51%, 0.15	p= 0.19
	Overall	3	RR 0.74 (0.62-0.88)	65%, 0.03	
	Within 1 week	2	RR 1.23 (0.43-5.52)	89%, 0.002	
Diabetes Mellitus	Within 2 weeks	2	RR 0.76 (0.42-1.38)	72%, 0.06	p= 0.44
	Overall	3	RR 0.90 (0.66-1.22)	81%, 0.001	

Supplementary Figure-S1. Flow chart presenting the selection of eligible studies.

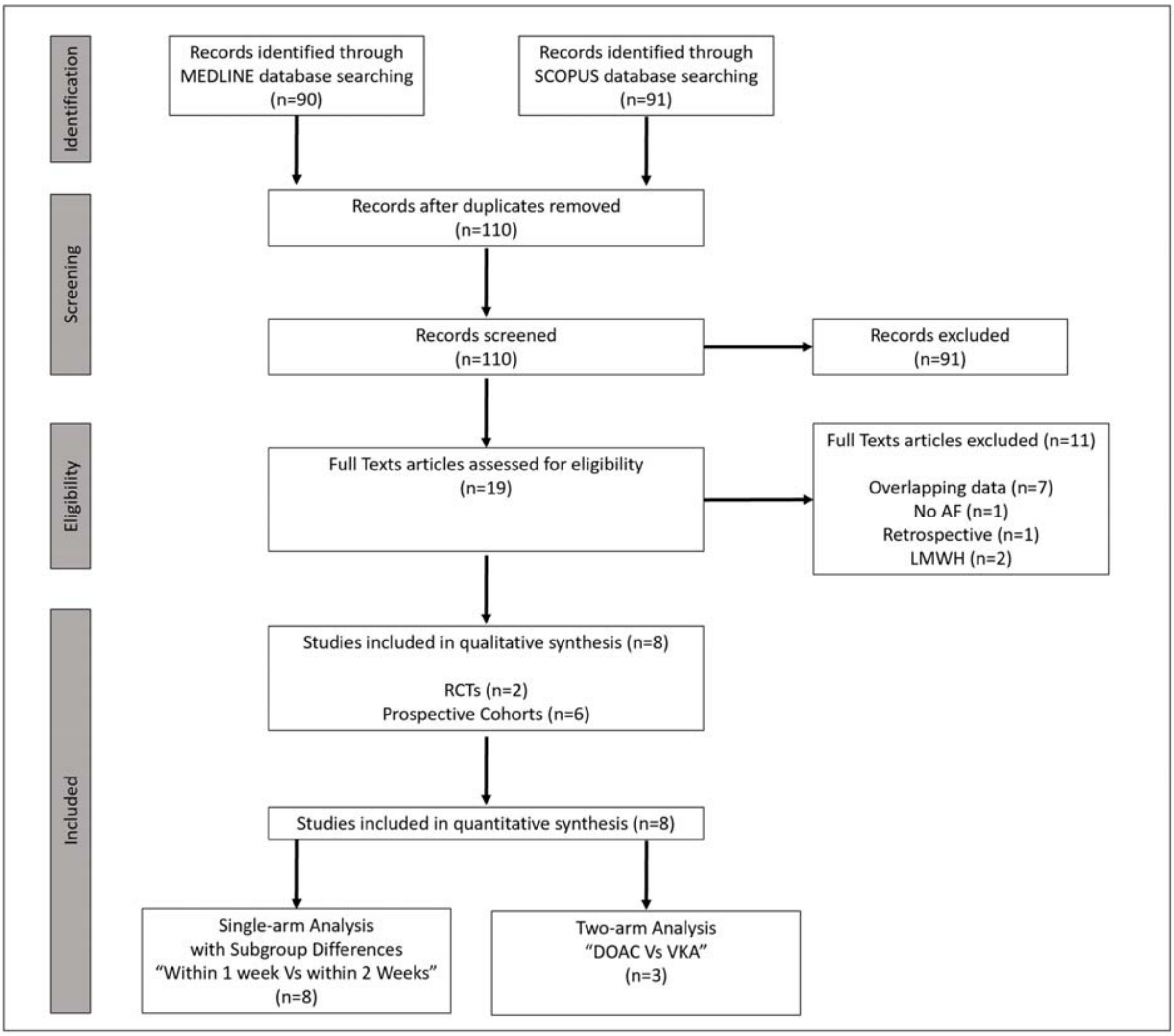

Supplementary Figure-S2. Risk of bias summary: review authors' judgments about each risk of bias item for each included study.

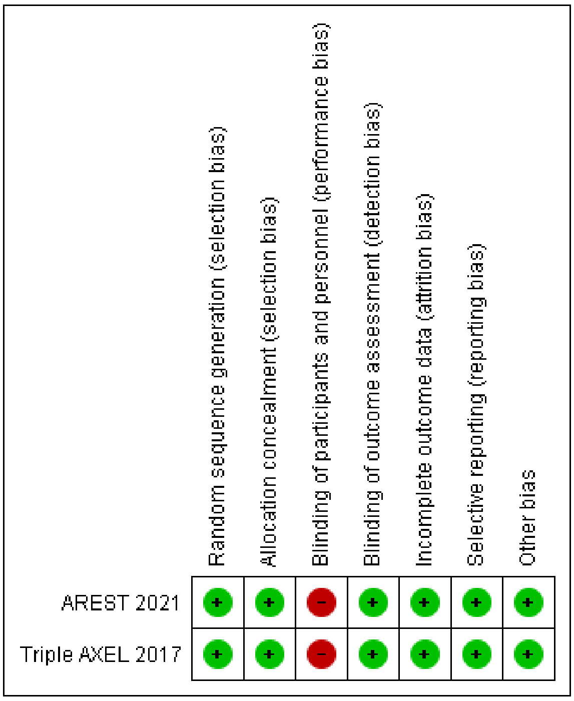

Supplementary Figure-S3. Risk of bias graph: review authors' judgments about each risk of bias item presented as percentages across all included studies.

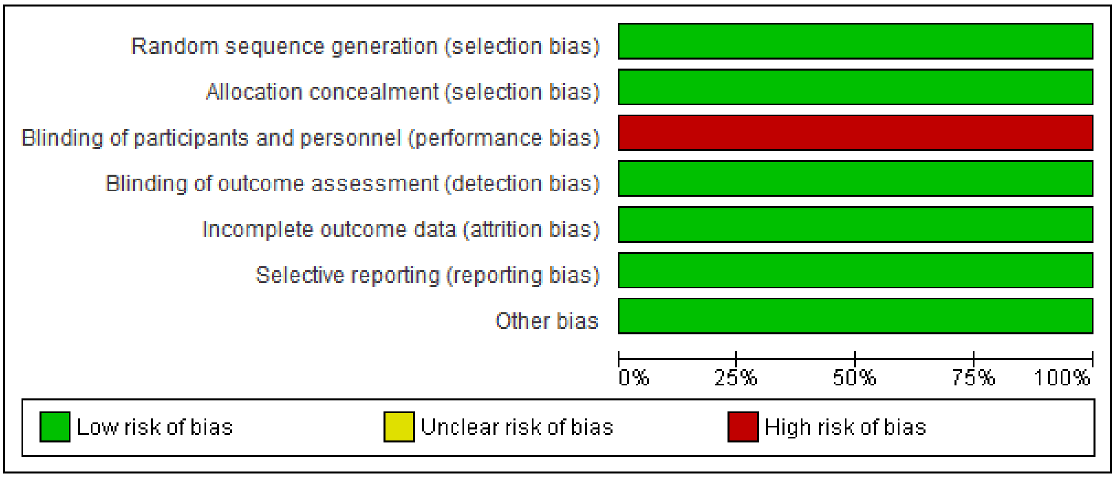

Supplementary Figure-S4. Forest plot presenting the pooled proportion of patients with symptomatic ICH following the initiation of oral anticoagulants.

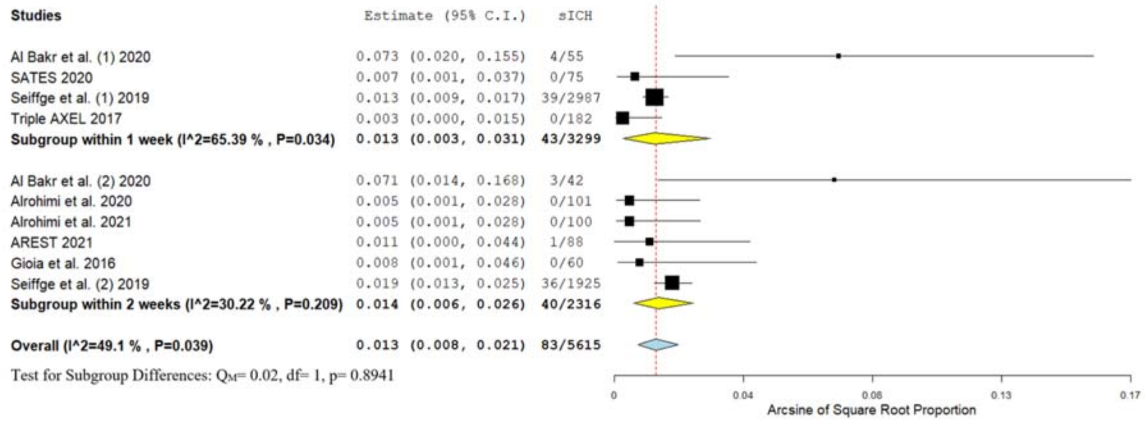

Supplementary Figure-S5. Forest plot presenting the pooled proportion of patients with any ICH following the initiation of oral anticoagulants.

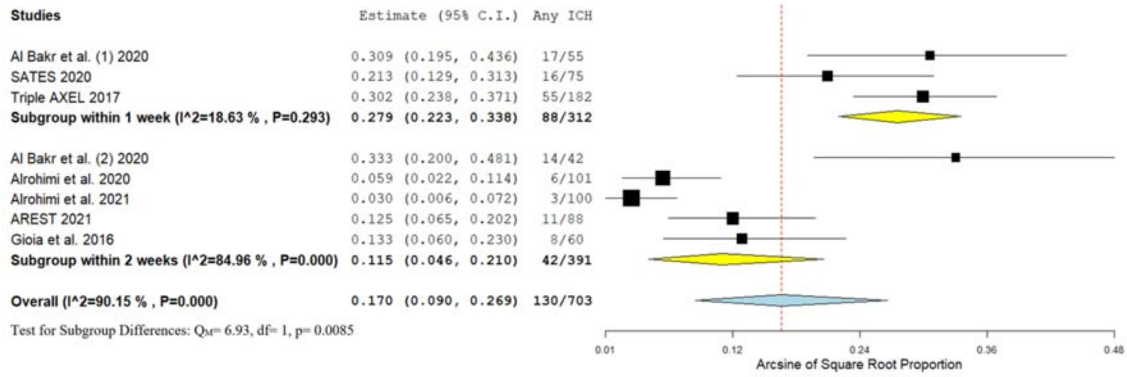

Supplementary Figure-S6. Forest plot presenting the pooled proportion of patients with all-cause mortality following the initiation of oral anticoagulants.

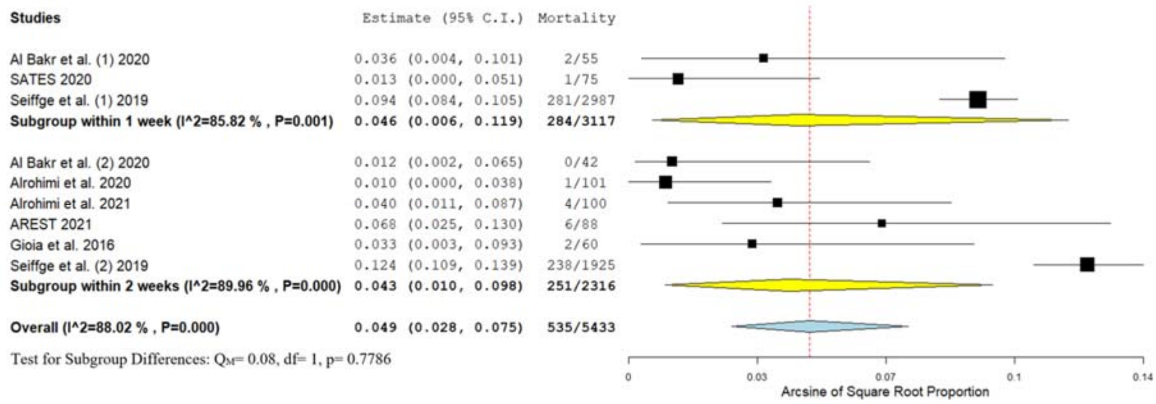

Supplementary Figure-S7. Forest plot presenting the pooled proportion of women initiating oral anticoagulants.

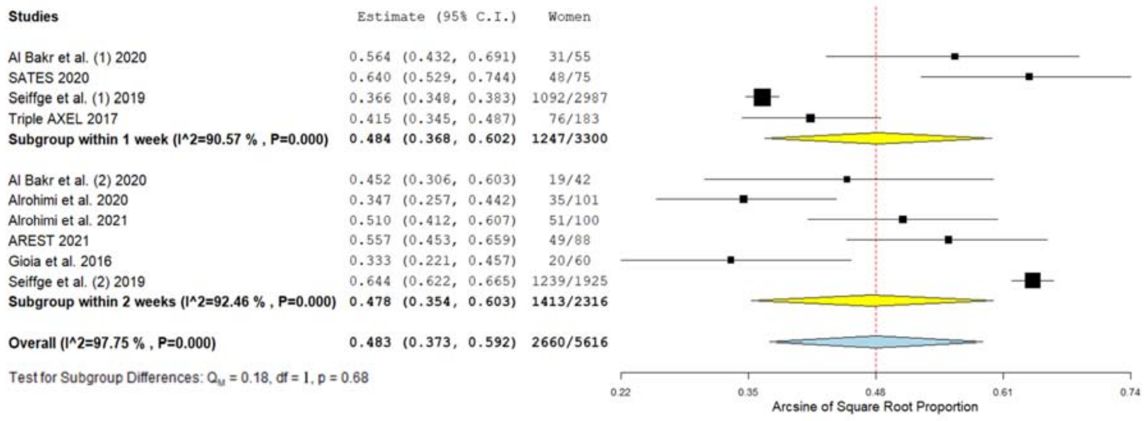

Supplementary Figure-S8. Forest plot presenting the mean age of patients initiating oral anticoagulants.

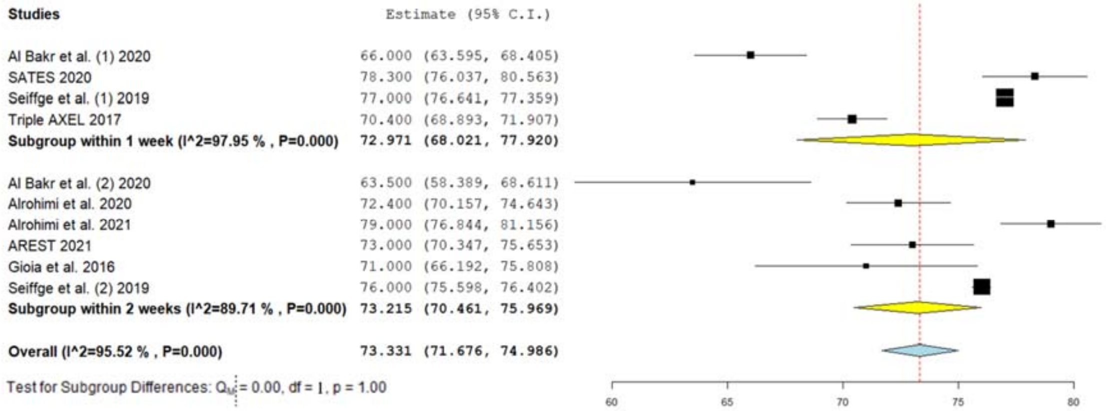

Supplementary Figure-S9. Forest plot presenting the mean baseline NIHSS score of patients initiating oral anticoagulants.

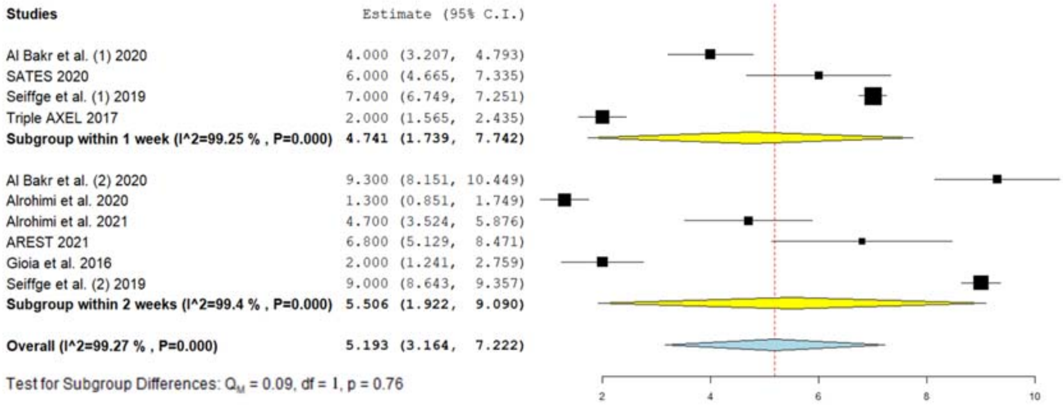

Supplementary Figure-S10. Forest plot presenting the mean baseline CHA_2DS_2-VASc score of patients initiating oral anticoagulants.

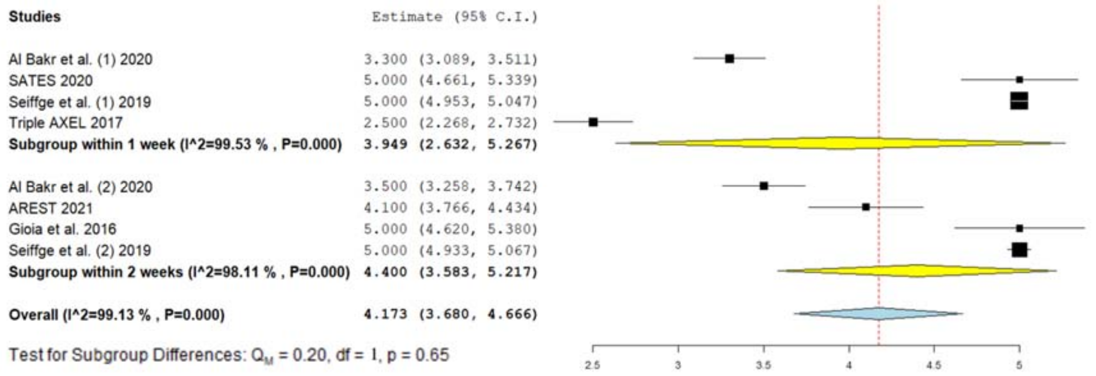

Supplementary Figure-S11. Forest plot presenting the mean baseline HAS-BLED score of patients initiating oral anticoagulants.

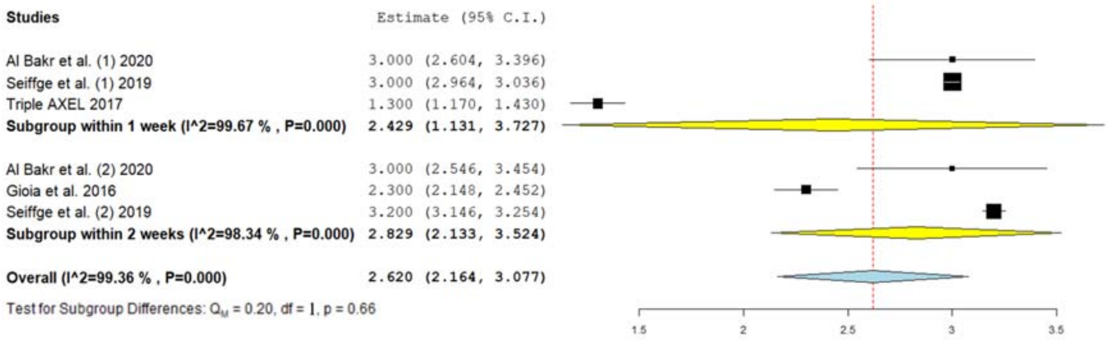

Supplementary Figure-S12. Forest plot presenting the mean baseline infarct volume (in ml) of patients initiating oral anticoagulants.

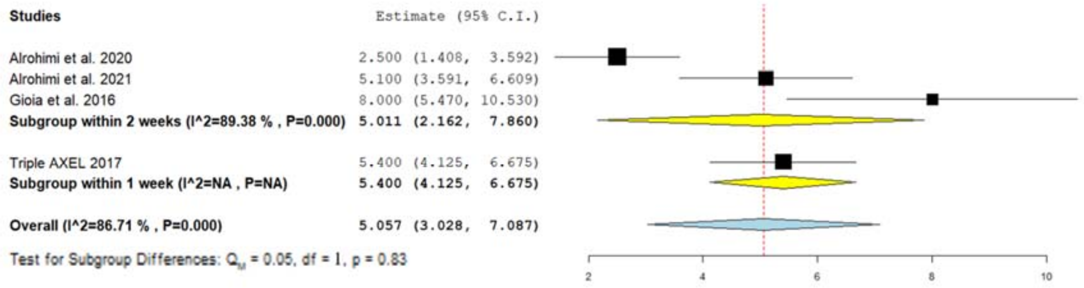

Supplementary Figure-S13. Forest plot presenting the pooled proportion of patients with a history of stroke prior the index event, initiating oral anticoagulants.

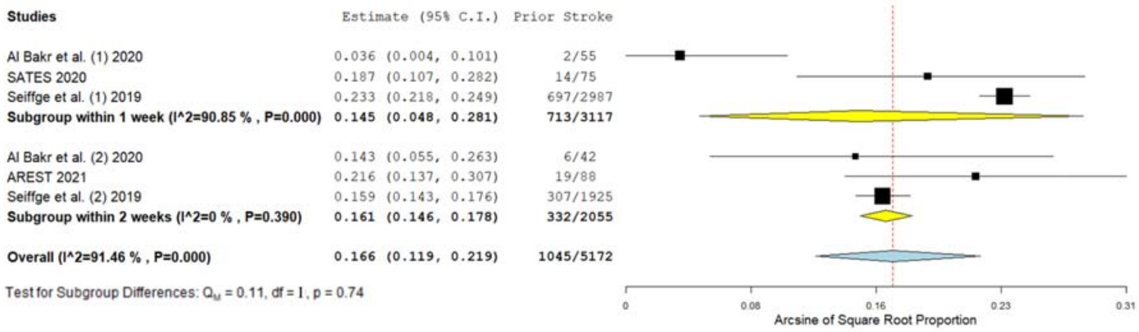

Supplementary Figure-S14. Forest plot presenting the pooled proportion of patients with hypertension, initiating oral anticoagulants.

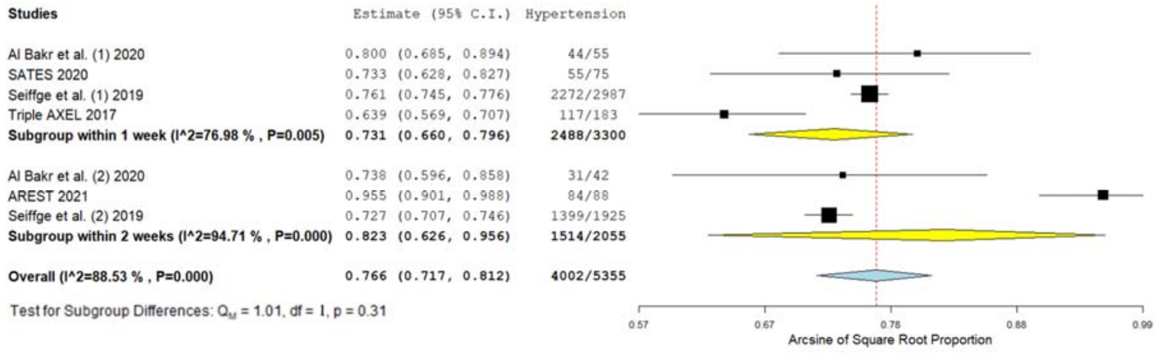

Supplementary Figure-S15. Forest plot presenting the pooled proportion of patients with dyslipidemia, initiating oral anticoagulants.

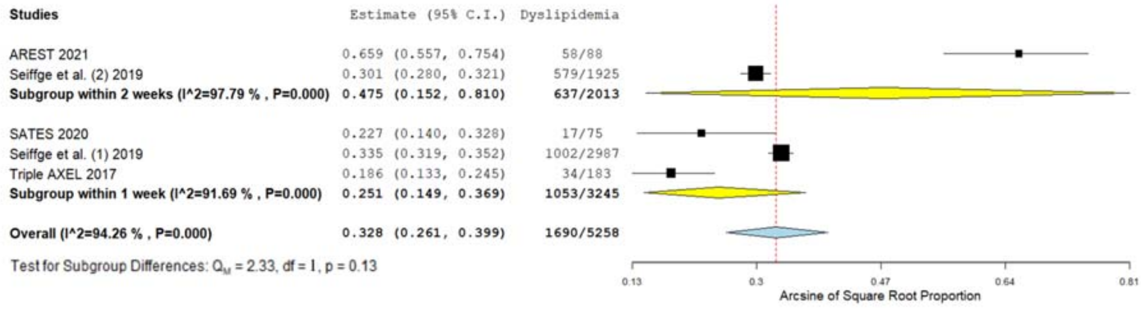

Supplementary Figure-S16. Forest plot presenting the pooled proportion of patients with diabetes mellitus, initiating oral anticoagulants.

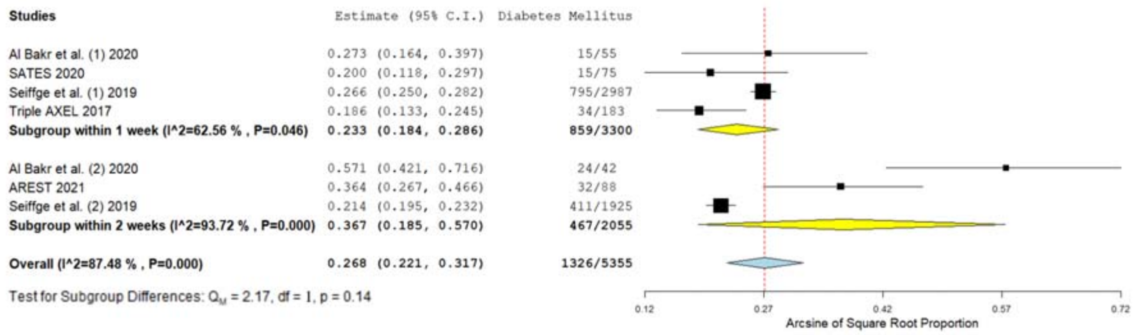

Supplementary Figure-S17. Forest plot presenting the pooled proportion of patients with chronic kidney failure, initiating oral anticoagulants.

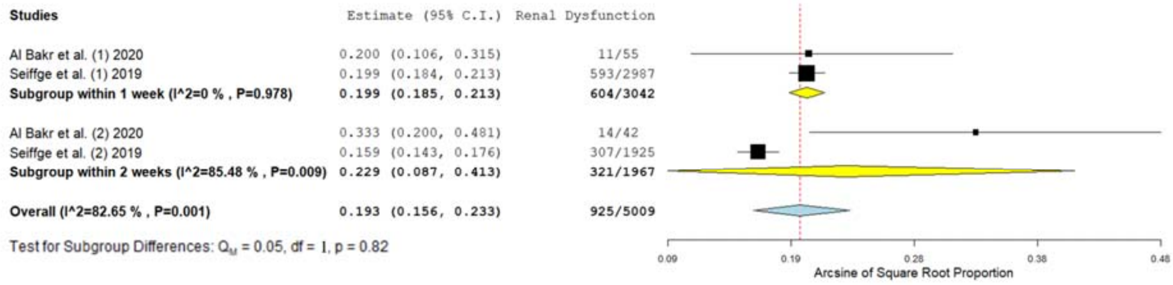

Supplementary Figure-S18. Funnel plot on the reported rates of ischemic stroke recurrence in patients with early initiation of oral anticoagulants (p for Egger's test= 0.8507).

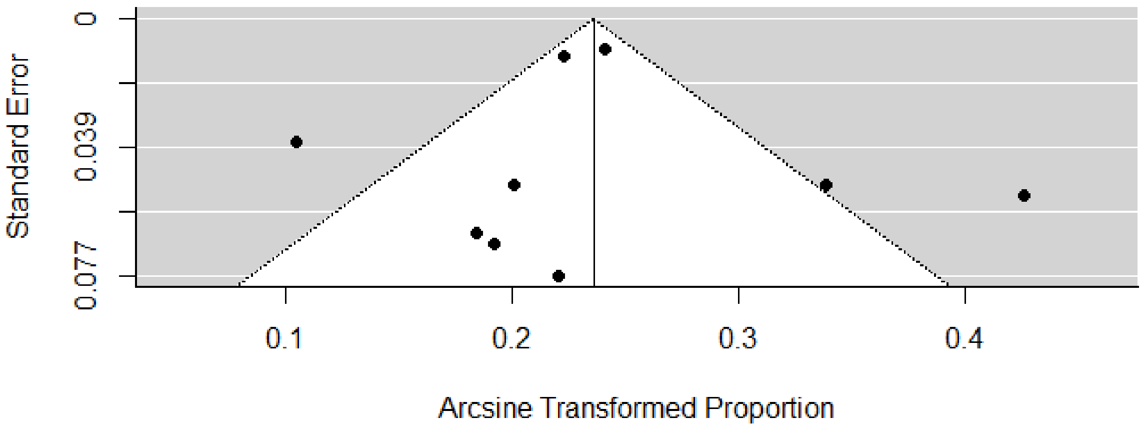

Supplementary Figure-S19. Funnel plot on the reported rates of symptomatic ICH in patients with early initiation of oral anticoagulants (p for Egger's test= 0.7688).

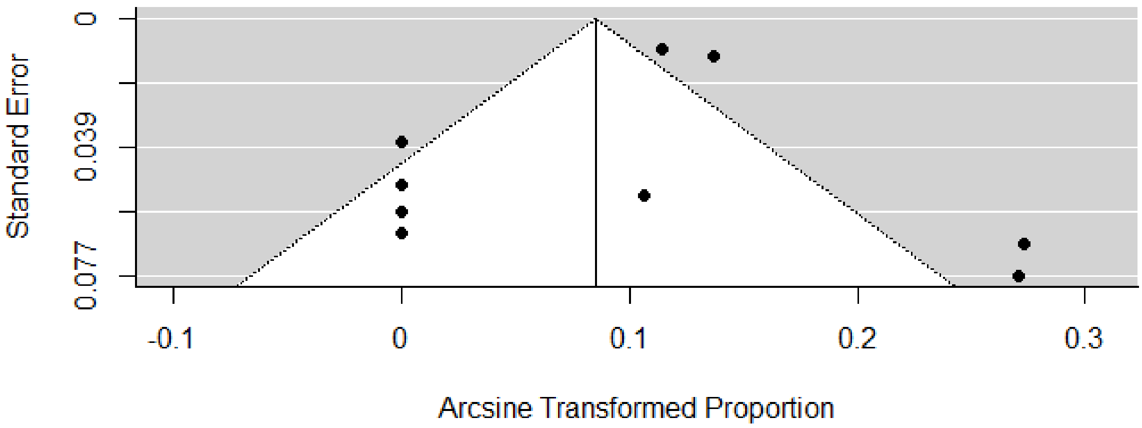

Supplementary Figure-S20. Funnel plot on the reported rates of any ICH in patients with early initiation of oral anticoagulants (p for Egger's test= 0.3874).

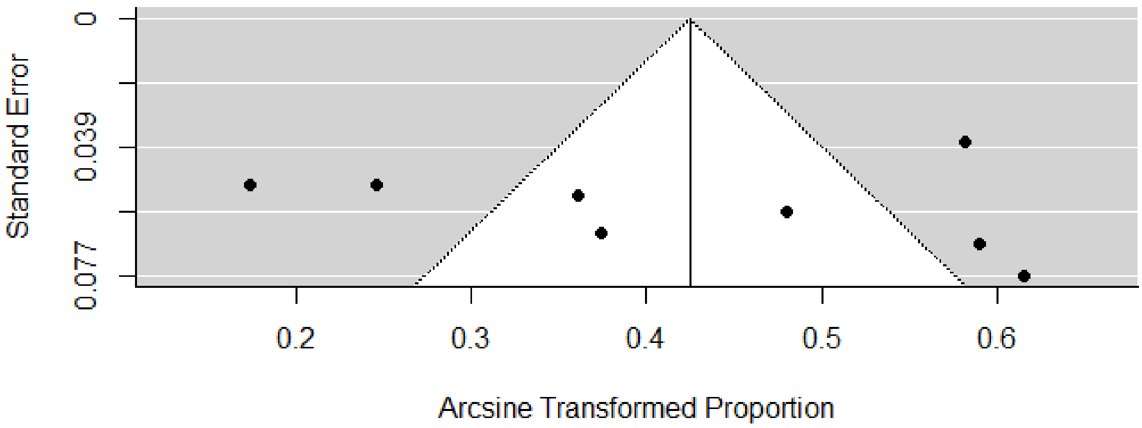

Supplementary Figure-S21. Funnel plot on the reported rates of all-cause mortality in patients with early initiation of oral anticoagulants (p for Egger's test< 0.001).

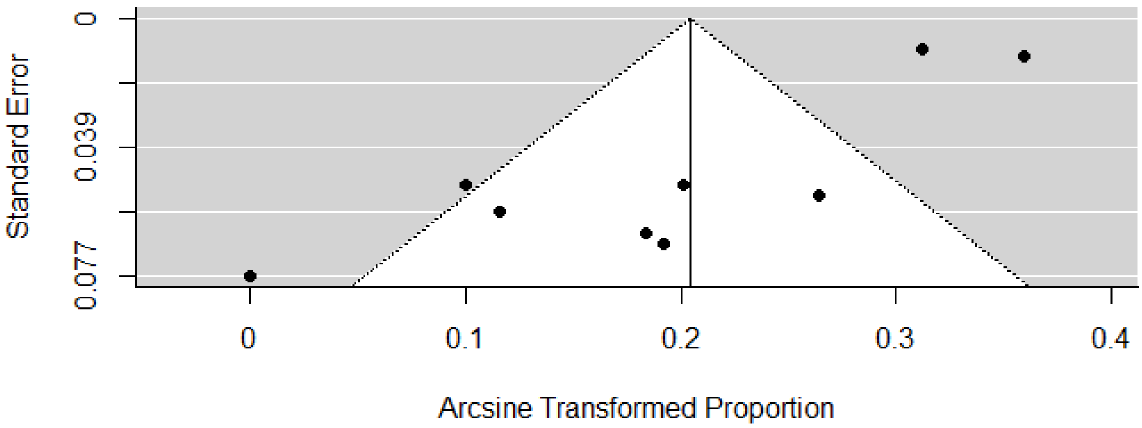

Supplementary Figure-S22. Forest plot presenting the risk ratio of symptomatic ICH among patients treated with DOACs versus VKAs, stratified by the timing of treatment initiation.

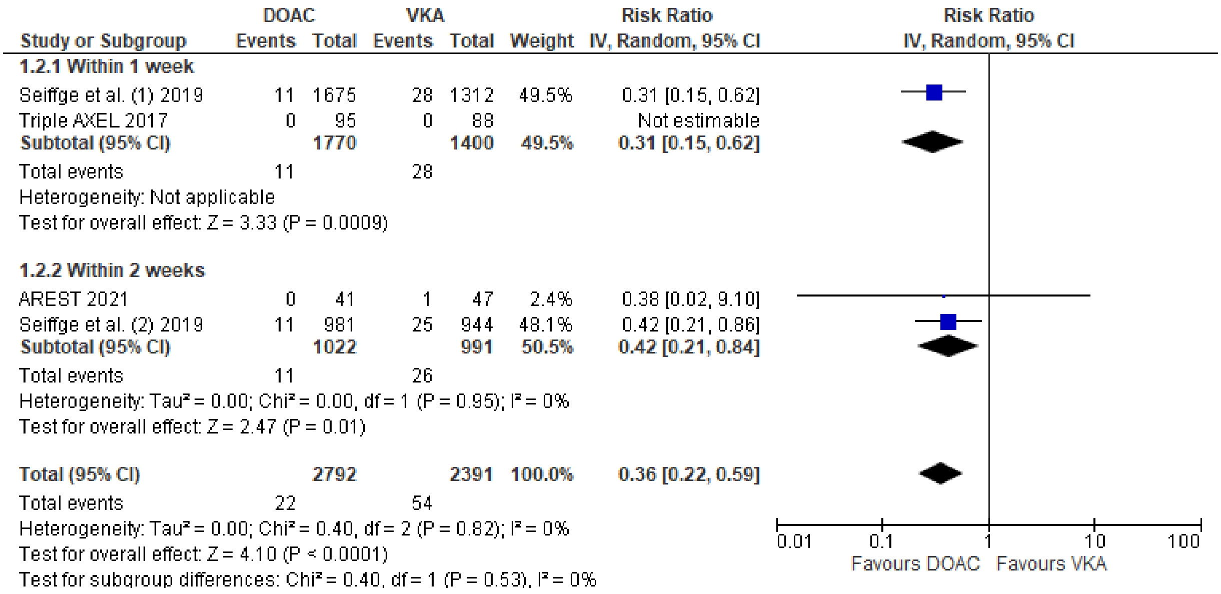

Supplementary Figure-S23. Forest plot presenting the risk ratio of any ICH among patients treated with DOACs versus VKAs, stratified by the timing of treatment initiation.

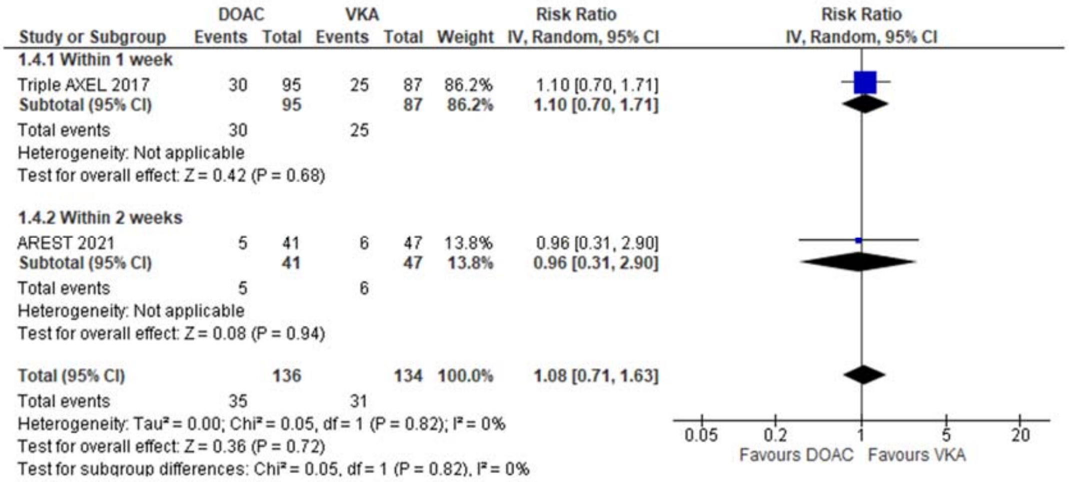

Supplementary Figure-S24. Forest plot presenting the risk ratio of all-cause mortality among patients treated with DOACs versus VKAs, stratified by the timing of treatment initiation.

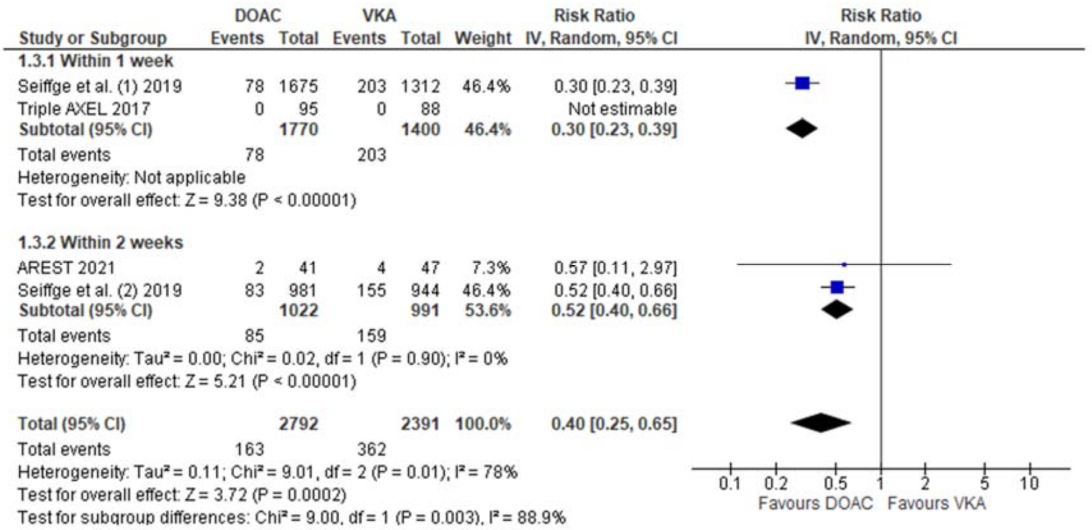

Supplementary Figure-S25. Forest plot presenting the risk ratio of women among patients treated with DOACs versus VKAs, stratified by the timing of treatment initiation.

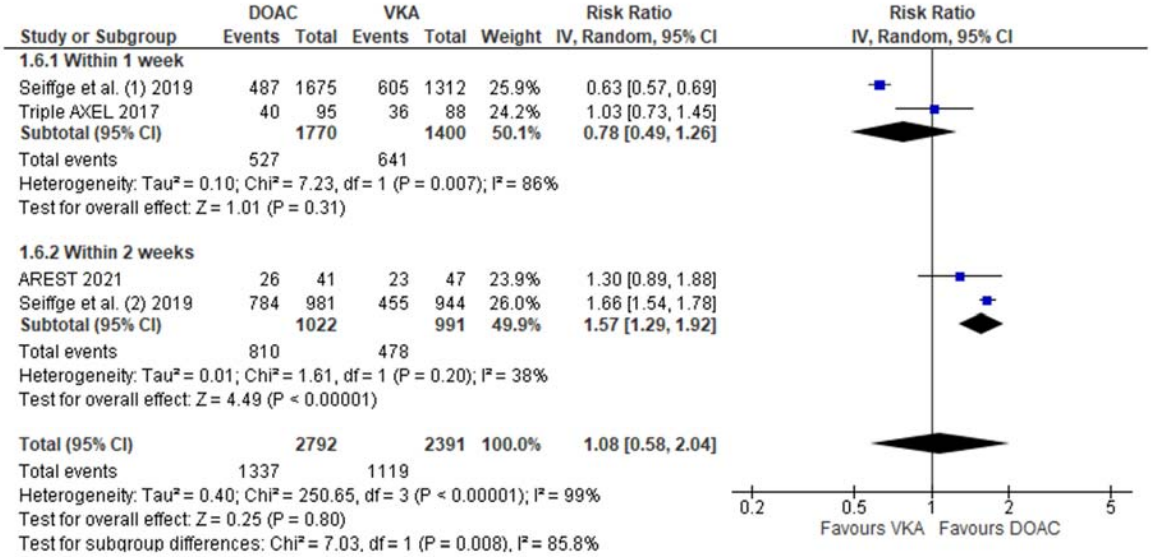

Supplementary Figure-S26. Forest plot presenting the mean difference of age (in years) among patients treated with DOACs versus VKAs, stratified by the timing of treatment initiation.

Study or Subgroup	DOAC Mean	DOAC SD	DOAC Total	VKA Mean	VKA SD	VKA Total	Weight	Mean Difference IV, Random, 95% CI
**1.5.1 Within 1 week**								
Seiffge et al. (1) 2019	77	11	1675	77	12	1312	58.6%	0.00 [-0.84, 0.84]
Triple AXEL 2017	70.2	10.1	95	70.6	10.9	88	4.4%	-0.40 [-3.45, 2.65]
**Subtotal (95% CI)**			**1770**			**1400**	**63.0%**	**-0.03 [-0.83, 0.78]**
Heterogeneity: Tau² = 0.00; Chi² = 0.06, df = 1 (P = 0.80); I² = 0%								
Test for overall effect: Z = 0.07 (P = 0.95)								
**1.5.2 Within 2 weeks**								
AREST 2021	72.6	14.9	41	74.3	10.5	47	1.4%	-1.70 [-7.16, 3.76]
Seiffge et al. (2) 2019	76	12	981	76	12	944	35.6%	0.00 [-1.07, 1.07]
**Subtotal (95% CI)**			**1022**			**991**	**37.0%**	**-0.06 [-1.12, 0.99]**
Heterogeneity: Tau² = 0.00; Chi² = 0.36, df = 1 (P = 0.55); I² = 0%								
Test for overall effect: Z = 0.12 (P = 0.91)								
**Total (95% CI)**			**2792**			**2391**	**100.0%**	**-0.04 [-0.68, 0.60]**
Heterogeneity: Tau² = 0.00; Chi² = 0.42, df = 3 (P = 0.94); I² = 0%								
Test for overall effect: Z = 0.13 (P = 0.90)								
Test for subgroup differences: Chi² = 0.00, df = 1 (P = 0.96), I² = 0%								

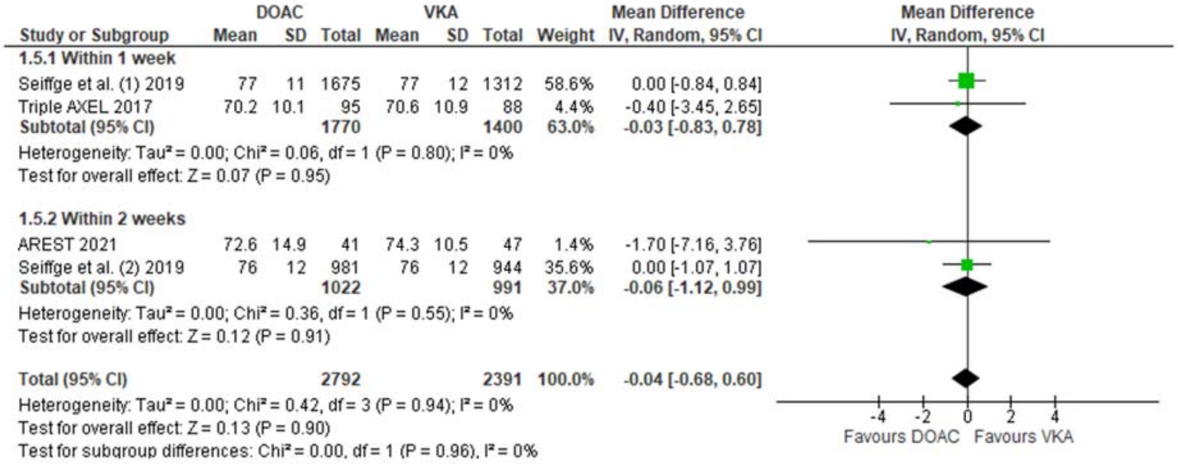

Supplementary Figure-S27. Forest plot presenting the mean difference of baseline NIHSS score among patients treated with DOACs versus VKAs, stratified by the timing of treatment initiation.

Study or Subgroup	DOAC Mean	DOAC SD	DOAC Total	VKA Mean	VKA SD	VKA Total	Weight	Mean Difference IV, Random, 95% CI
**1.7.1 Within 1 week**								
Seiffge et al. (1) 2019	6	6	1675	8	7	1312	30.9%	-2.00 [-2.48, -1.52]
Triple AXEL 2017	3.4	4.6	95	2	3	88	27.0%	1.40 [0.28, 2.52]
**Subtotal (95% CI)**			**1770**			**1400**	**57.9%**	**-0.34 [-3.67, 2.99]**
Heterogeneity: Tau² = 5.59; Chi² = 30.12, df = 1 (P < 0.00001); I² = 97%								
Test for overall effect: Z = 0.20 (P = 0.84)								
**1.7.2 Within 2 weeks**								
AREST 2021	6.5	7.6	41	7.2	8.4	47	12.2%	-0.70 [-4.04, 2.64]
Seiffge et al. (2) 2019	8	7	981	10	8	944	29.9%	-2.00 [-2.67, -1.33]
**Subtotal (95% CI)**			**1022**			**991**	**42.1%**	**-1.95 [-2.61, -1.29]**
Heterogeneity: Tau² = 0.00; Chi² = 0.56, df = 1 (P = 0.46); I² = 0%								
Test for overall effect: Z = 5.79 (P < 0.00001)								
**Total (95% CI)**			**2792**			**2391**	**100.0%**	**-0.92 [-2.41, 0.56]**
Heterogeneity: Tau² = 1.80; Chi² = 32.03, df = 3 (P < 0.00001); I² = 91%								
Test for overall effect: Z = 1.22 (P = 0.22)								
Test for subgroup differences: Chi² = 0.86, df = 1 (P = 0.35), I² = 0%								

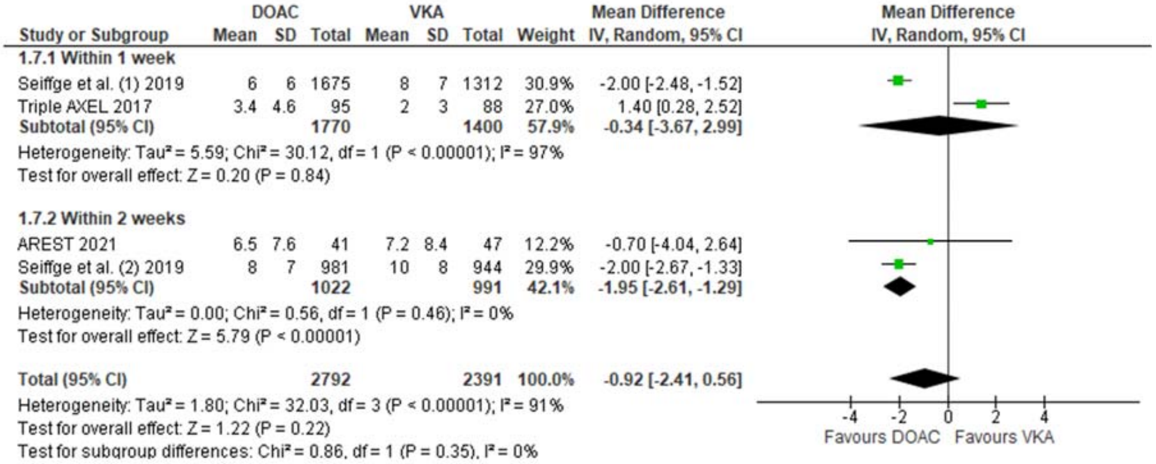

Supplementary Figure-S28. Forest plot presenting the mean difference of baseline CHA_2DS_2-VASc score among patients treated with DOACs versus VKAs, stratified by the timing of treatment initiation.

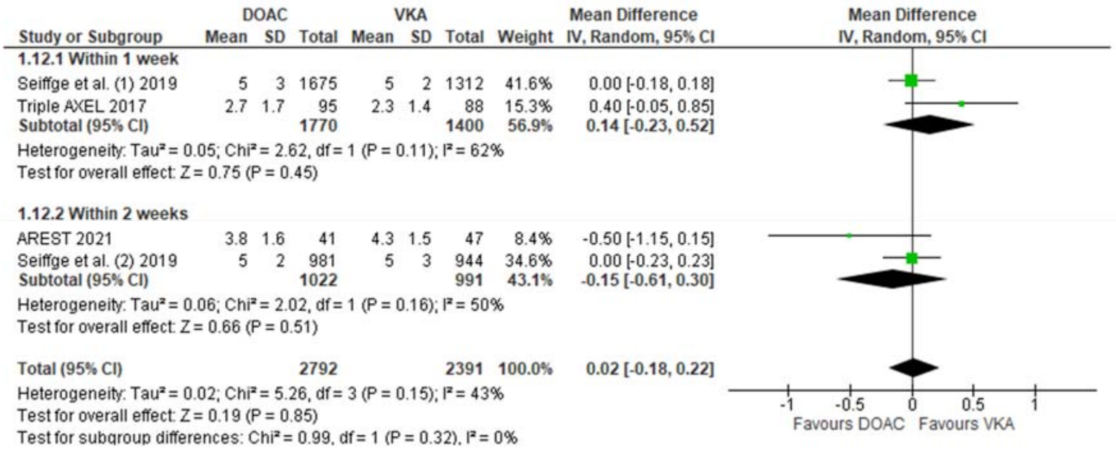

Supplementary Figure-S29. Forest plot presenting the mean difference of baseline HAS-BLED score among patients treated with DOACs versus VKAs, stratified by the timing of treatment initiation.

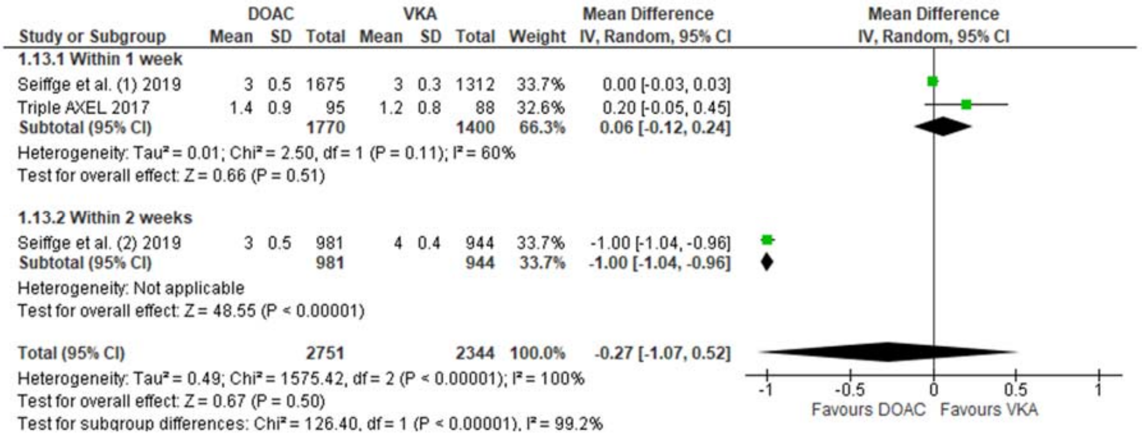

Supplementary Figure-S30. Forest plot presenting the risk ratio of prior stroke among patients treated with DOACs versus VKAs, stratified by the timing of treatment initiation.

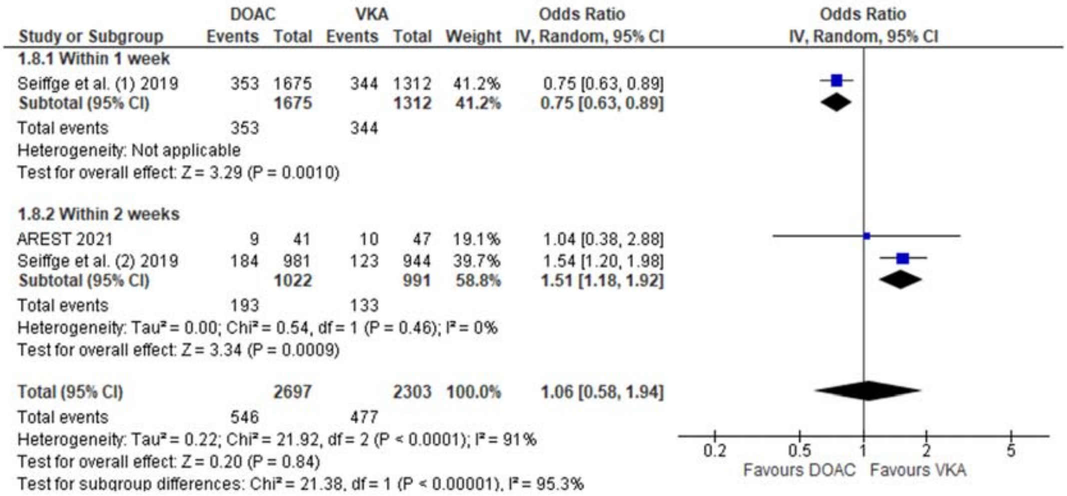

Supplementary Figure-S31. Forest plot presenting the risk ratio of hypertension among patients treated with DOACs versus VKAs, stratified by the timing of treatment initiation.

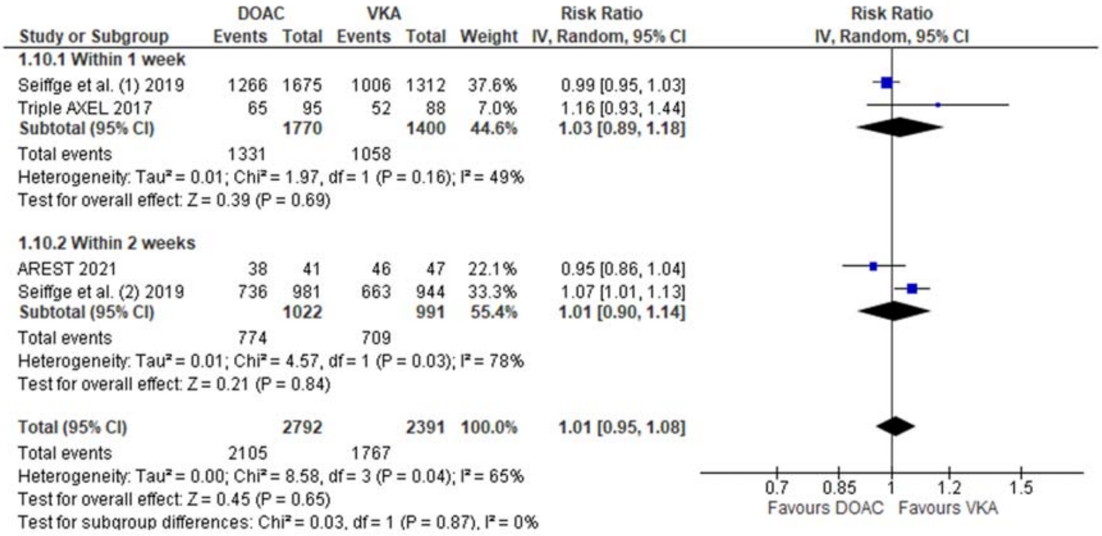

Supplementary Figure-S32. Forest plot presenting the risk ratio of dyslipidemia among patients treated with DOACs versus VKAs, stratified by the timing of treatment initiation.

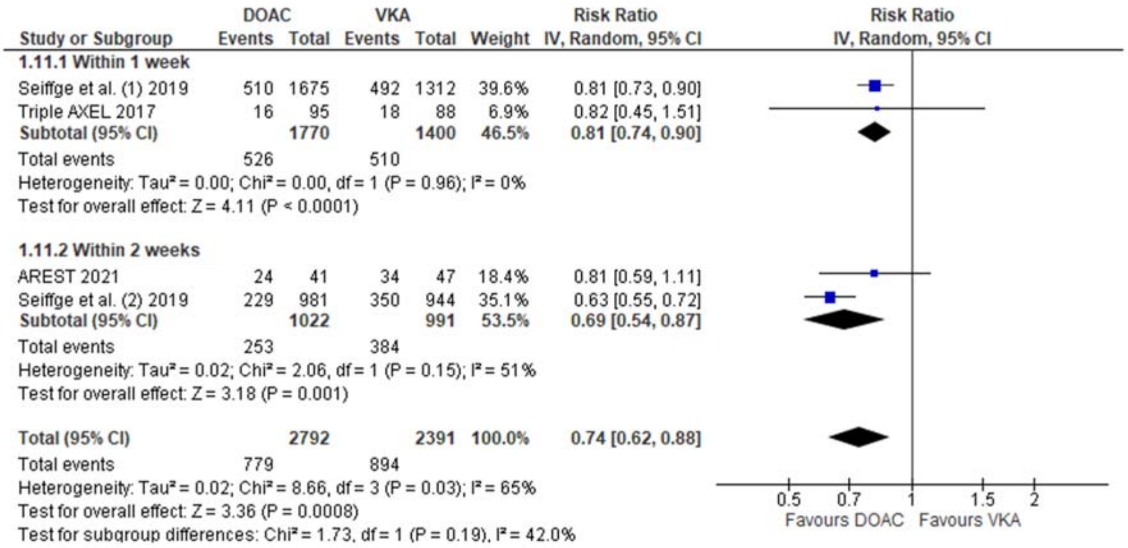

Supplementary Figure-S33. Forest plot presenting the risk ratio of diabetes mellitus among patients treated with DOACs versus VKAs, stratified by the timing of treatment initiation.

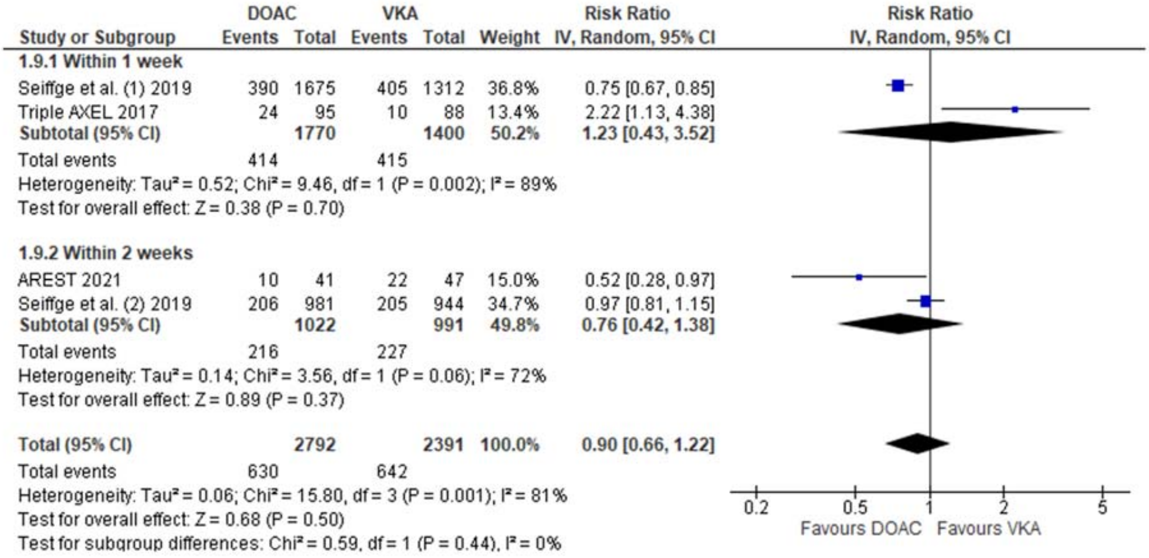

Supplementary Figure-S34. Forest plot of leave-one-out analysis for the pooled proportion of patients with IS recurrence following the initiation of oral anticoagulants.

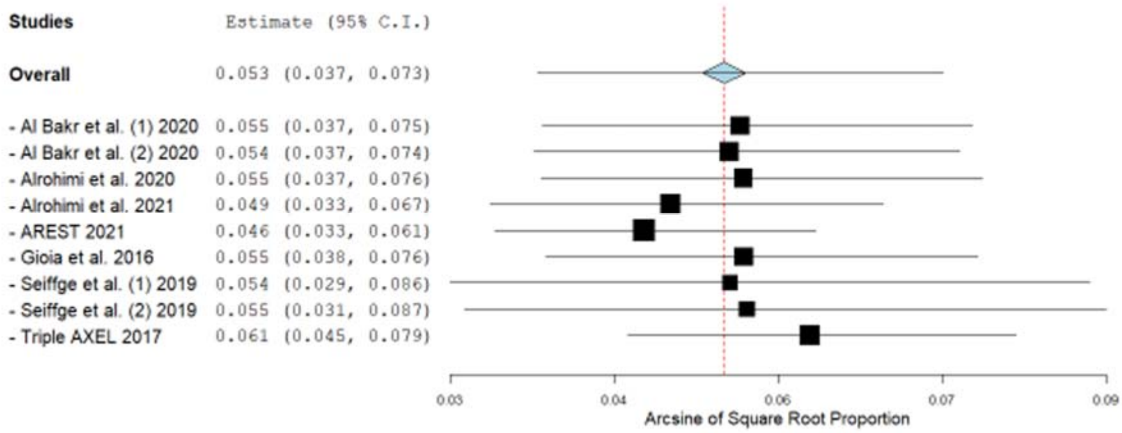

Supplementary Figure-S35. Forest plot of leave-one-out analysis for the pooled proportion of patients with symptomatic ICH following the initiation of oral anticoagulants.

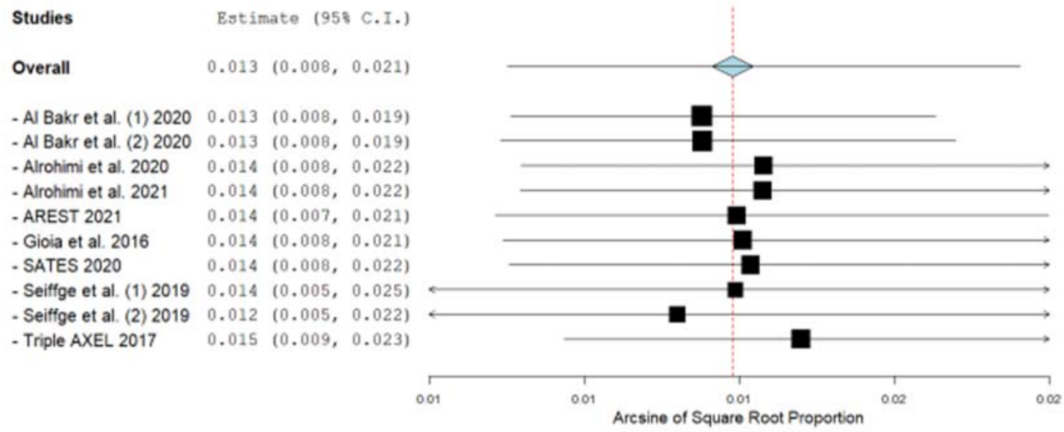

Supplementary Figure-S36. Forest plot of leave-one-out analysis for the pooled proportion of patients with any ICH following the initiation of oral anticoagulants.

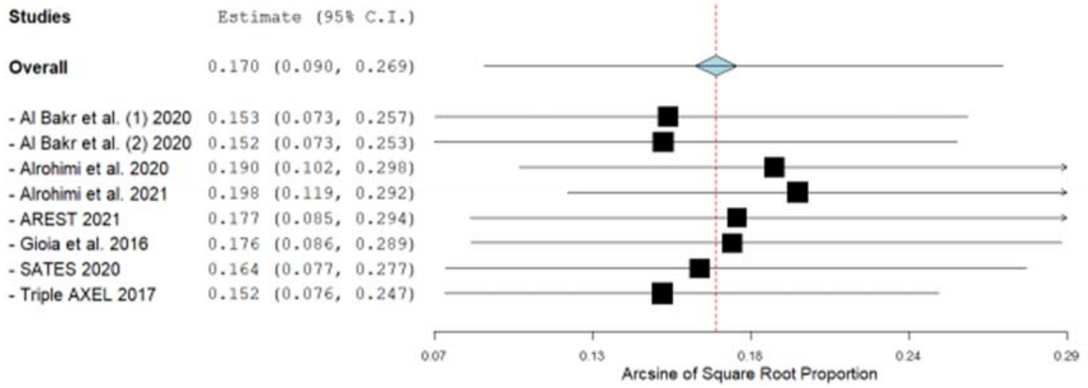

Supplementary Figure-S37. Forest plot of leave-one-out analysis for the pooled proportion of patients with all-cause mortality following the initiation of oral anticoagulants.

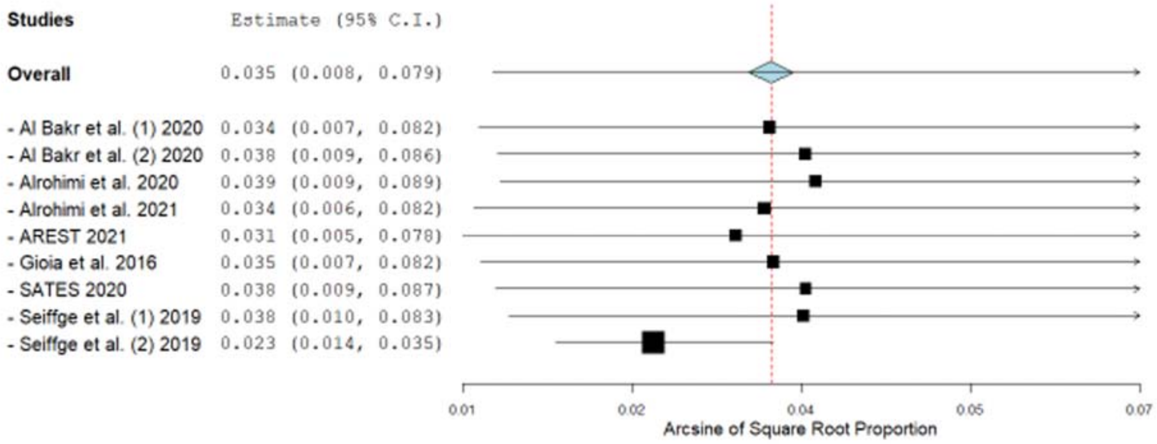

Supplementary Figure-S38. Forest plot of leave-one-out analysis for the risk ratio of IS recurrence among patients treated with DOACs versus VKAs.

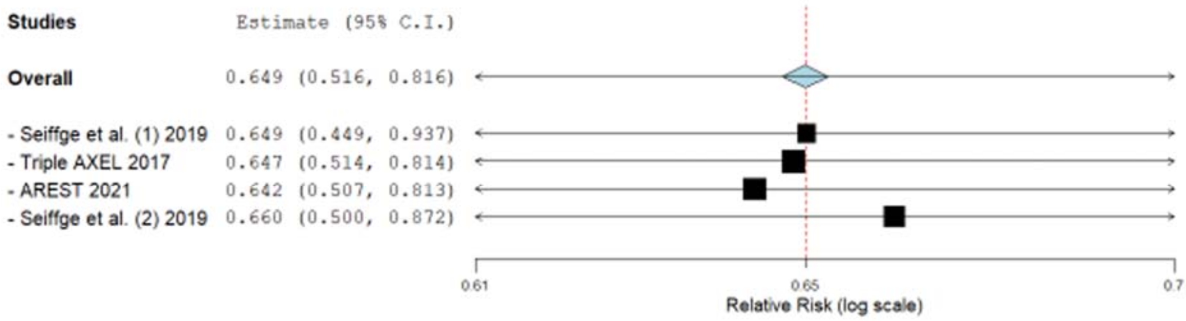

Supplementary Figure-S39. Forest plot of leave-one-out analysis for the risk ratio of symptomatic ICH among patients treated with DOACs versus VKAs.

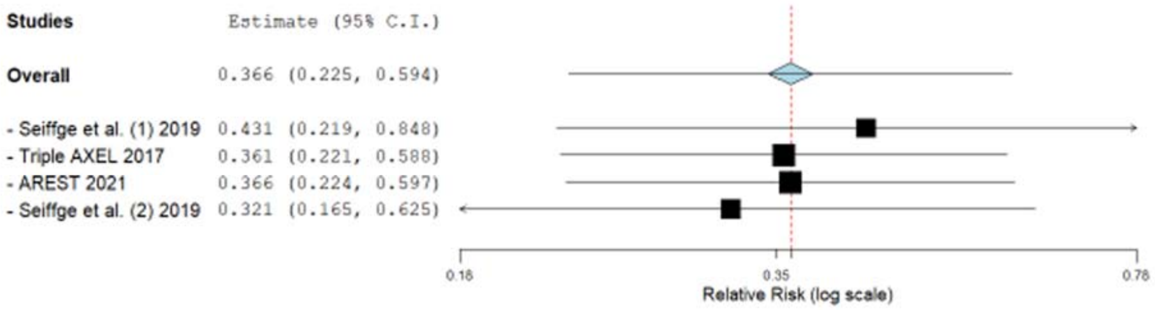

Supplementary Figure-S40. Forest plot of leave-one-out analysis for the risk ratio of any ICH among patients treated with DOACs versus VKAs.

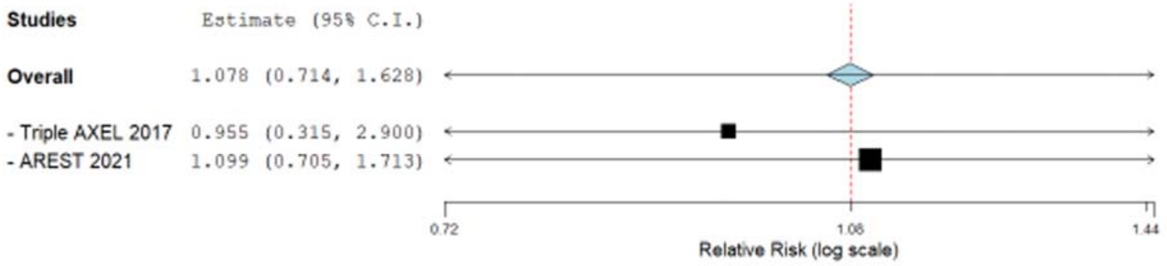

Supplementary Figure-S41. Forest plot of leave-one-out analysis for the risk ratio of all-cause mortality among patients treated with DOACs versus VKAs.

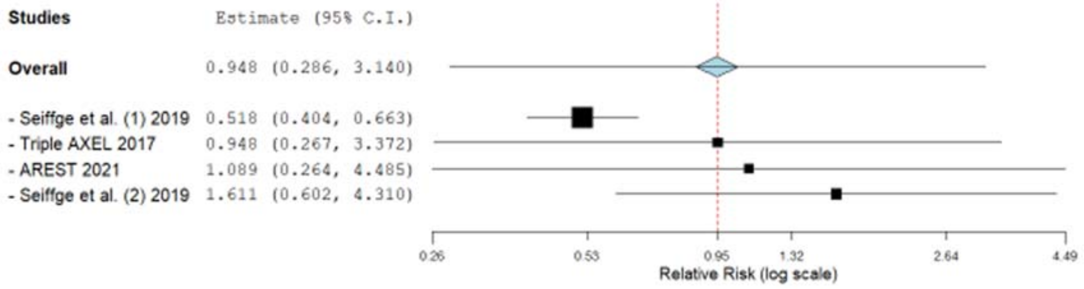

Supplementary Figure-S42. Forest plot presenting the pooled proportion of patients with IS recurrence following the initiation of oral anticoagulants, stratified by study design (observational studies versus RCTs).

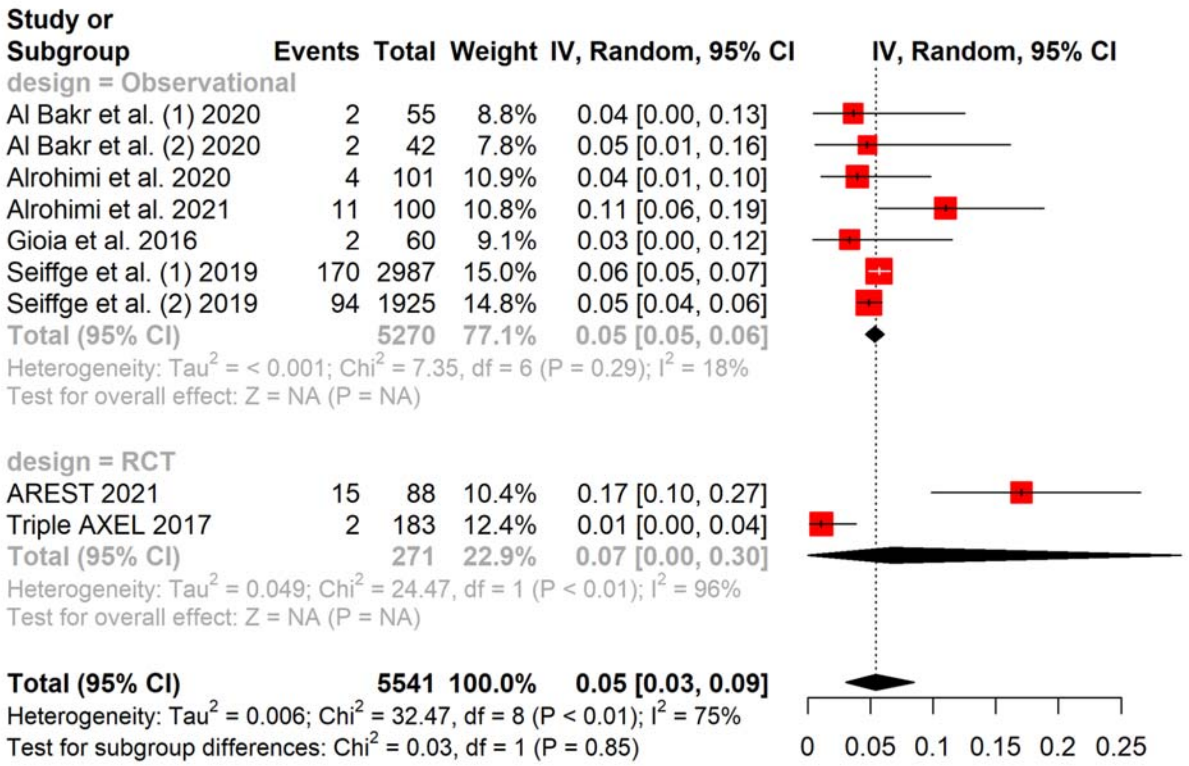

Supplementary Figure-S43. Forest plot presenting the pooled proportion of patients with symptomatic ICH following the initiation of oral anticoagulants, stratified by study design (observational studies versus RCTs).

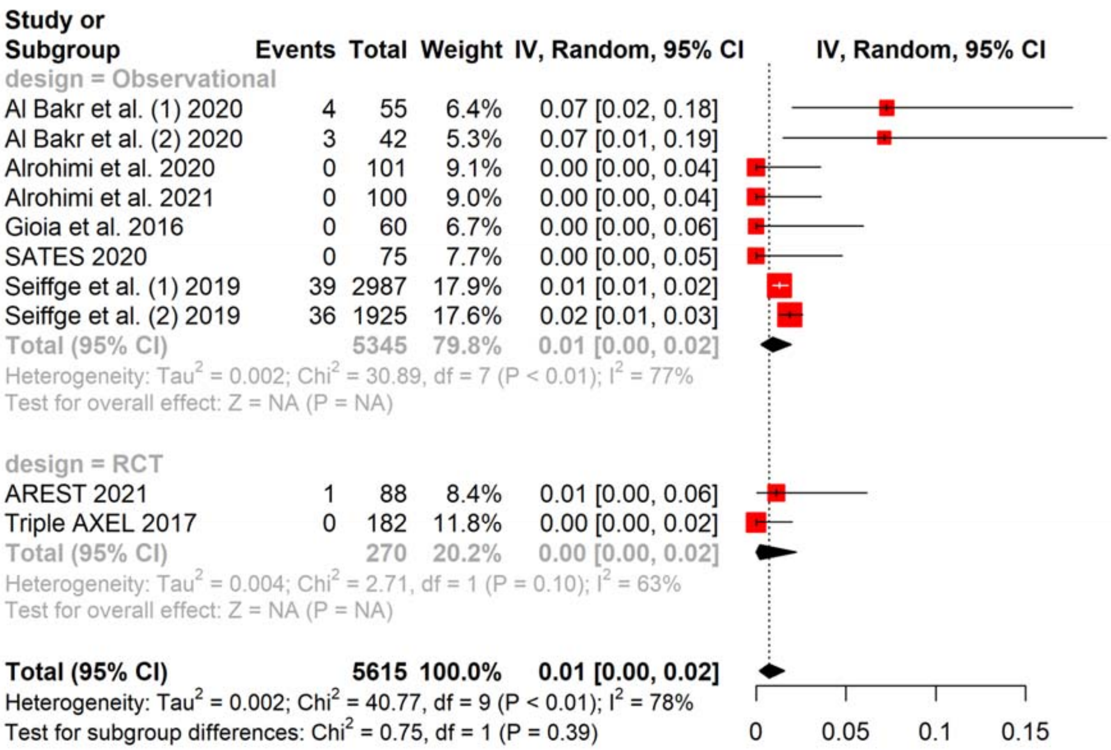

Supplementary Figure-S44. Forest plot presenting the pooled proportion of patients with any ICH following the initiation of oral anticoagulants, stratified by study design (observational studies versus RCTs).

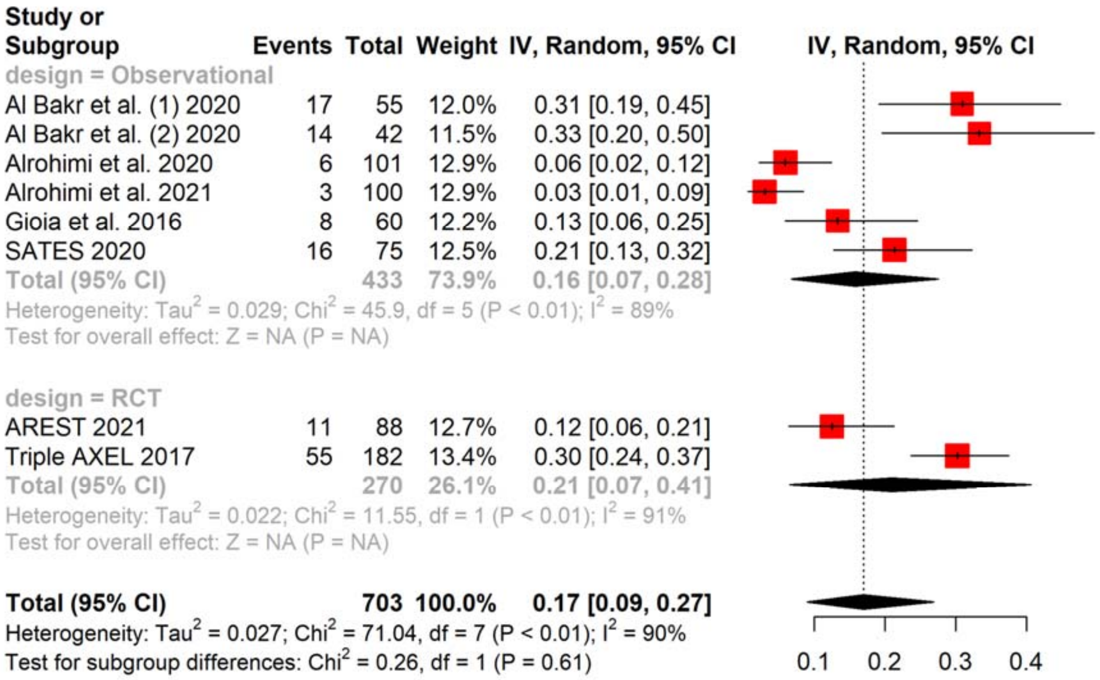

Supplementary Figure-S45. Forest plot presenting the pooled proportion of patients with all-cause mortality ICH following the initiation of oral anticoagulants, stratified by study design (observational studies versus RCTs).

Study or Subgroup	Events	Total	Weight	IV, Random, 95% CI
design = Observational				
Al Bakr et al. (1) 2020	2	55	8.8%	0.04 [0.00, 0.13]
Al Bakr et al. (2) 2020	0	42	7.7%	0.00 [0.00, 0.08]
Alrohimi et al. 2020	1	101	11.1%	0.01 [0.00, 0.05]
Alrohimi et al. 2021	4	100	11.1%	0.04 [0.01, 0.10]
Gioia et al. 2016	2	60	9.1%	0.03 [0.00, 0.12]
SATES 2020	1	75	10.0%	0.01 [0.00, 0.07]
Seiffge et al. (1) 2019	281	2987	15.8%	0.09 [0.08, 0.11]
Seiffge et al. (2) 2019	238	1925	15.7%	0.12 [0.11, 0.14]
Total (95% CI)		5345	89.4%	0.04 [0.02, 0.07]
Heterogeneity: $Tau^2 = 0.006$; $Chi^2 = 75.2$, df = 7 (P < 0.01); $I^2 = 91\%$				
Test for overall effect: Z = NA (P = NA)				
design = RCT				
AREST 2021	6	88	10.6%	0.07 [0.03, 0.14]
Test for overall effect: Z = NA (P = NA)				
**Total (95% CI)**		**5433**	**100.0%**	**0.04 [0.02, 0.07]**
Heterogeneity: $Tau^2 = 0.005$; $Chi^2 = 76.09$, df = 8 (P < 0.01); $I^2 = 89\%$				
Test for subgroup differences: $Chi^2 = 0.89$, df = 1 (P = 0.35)				

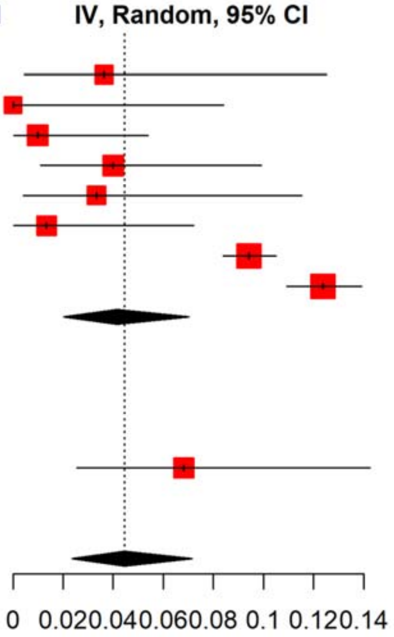

Supplementary Figure-S46. Forest plot presenting the risk ratio of IS recurrence among patients treated with DOACs versus VKAs, stratified by study design (observational studies versus RCTs).

Study or Subgroup	Experimental Events	Experimental Total	Control Events	Control Total	Weight	Risk Ratio IV, Random, 95% CI
design = Observational						
Seiffge et al. (1) 2019	77	1675	93	1312	61.2%	0.65 [0.48, 0.87]
Seiffge et al. (2) 2019	37	981	57	944	32.2%	0.62 [0.42, 0.94]
Total (95% CI)		2656		2256	93.4%	0.64 [0.50, 0.81]
Heterogeneity: $Tau^2 = 0$; $Chi^2 = 0.02$, df = 1 (P = 0.88); $I^2 = 0\%$						
Test for overall effect: Z = -3.68 (P < 0.01)						
design = RCT						
Triple AXEL 2017	1	95	1	88	0.7%	0.93 [0.06, 14.59]
AREST 2021	6	41	9	47	5.9%	0.76 [0.30, 1.96]
Total (95% CI)		136		135	6.6%	0.78 [0.32, 1.91]
Heterogeneity: $Tau^2 = 0$; $Chi^2 = 0.02$, df = 1 (P = 0.90); $I^2 = 0\%$						
Test for overall effect: Z = -0.55 (P = 0.59)						
**Total (95% CI)**		**2792**		**2391**	**100.0%**	**0.65 [0.52, 0.82]**
Heterogeneity: $Tau^2 = 0$; $Chi^2 = 0.21$, df = 3 (P = 0.98); $I^2 = 0\%$						
Test for overall effect: Z = -3.70 (P < 0.01)						
Test for subgroup differences: $Chi^2 = 0.18$, df = 1 (P = 0.68)						

Risk Ratio
IV, Random, 95% CI

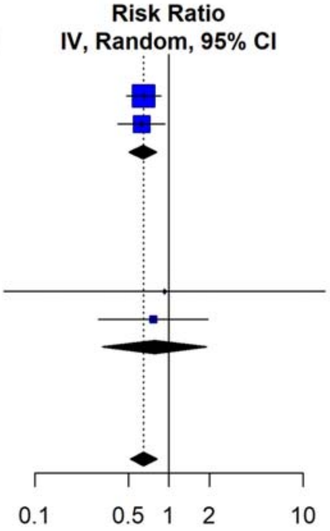

Supplementary Figure-S47. Forest plot presenting the risk ratio of symptomatic ICH among patients treated with DOACs versus VKAs, stratified by study design (observational studies versus RCTs).

Study or Subgroup	Experimental Events	Experimental Total	Control Events	Control Total	Weight	Risk Ratio IV, Random, 95% CI
design = Observational						
Seiffge et al. (1) 2019	11	1675	28	1312	49.5%	0.31 [0.15, 0.62]
Seiffge et al. (2) 2019	11	981	25	944	48.1%	0.42 [0.21, 0.86]
Total (95% CI)		2656		2256	97.6%	0.36 [0.22, 0.59]
Heterogeneity: $Tau^2 = 0$; $Chi^2 = 0.4$, df = 1 (P = 0.53); $I^2 = 0\%$						
Test for overall effect: Z = -4.05 (P < 0.01)						
design = RCT						
Triple AXEL 2017	0	95	0	88	0.0%	
AREST 2021	0	41	1	47	2.4%	0.38 [0.02, 9.10]
Test for overall effect: Z = -0.60 (P = 0.55)						
**Total (95% CI)**		**2792**		**2391**	**100.0%**	**0.36 [0.22, 0.59]**
Heterogeneity: $Tau^2 = 0$; $Chi^2 = 0.40$, df = 2 (P = 0.82); $I^2 = 0\%$						
Test for overall effect: Z = -4.10 (P < 0.01)						
Test for subgroup differences: $Chi^2 = 0.00$, df = 1 (P = 0.97)						

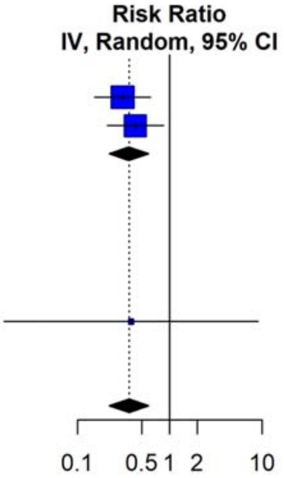

Supplementary Figure-S48. Forest plot presenting the risk ratio of any ICH among patients treated with DOACs versus VKAs, stratified by study design (observational studies versus RCTs). In the present analysis, only RCTs were included.

Study or Subgroup	Experimental Events	Experimental Total	Control Events	Control Total	Weight	Risk Ratio IV, Random, 95% CI
design = RCT						
Triple AXEL 2017	30	95	25	87	86.2%	1.10 [0.70, 1.71]
AREST 2021	5	41	6	47	13.8%	0.96 [0.31, 2.90]
Total (95% CI)		136		134	100.0%	1.08 [0.71, 1.63]
Heterogeneity: Tau^2 = 0; Chi^2 = 0.05, df = 1 (P = 0.82); I^2 = 0%						
Test for overall effect: Z = 0.36 (P = 0.72)						
**Total (95% CI)**		**136**		**134**	**100.0%**	**1.08 [0.71, 1.63]**
Heterogeneity: Tau^2 = 0; Chi^2 = 0.05, df = 1 (P = 0.82); I^2 = 0%						
Test for overall effect: Z = 0.36 (P = 0.72)						
Test for subgroup differences: Chi^2 = 0.00, df = 0 (P = NA)						

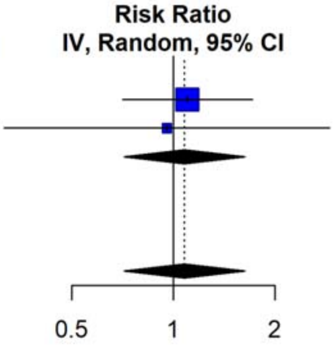

Supplementary Figure-S49. Forest plot presenting the risk ratio of all-cause mortality among patients treated with DOACs versus VKAs, stratified by study design (observational studies versus RCTs).

Study or Subgroup	Experimental Events	Experimental Total	Control Events	Control Total	Weight	Risk Ratio IV, Random, 95% CI
design = Observational						
Seiffge et al. (1) 2019	78	1675	203	1312	46.4%	0.30 [0.23, 0.39]
Seiffge et al. (2) 2019	83	981	155	944	46.4%	0.52 [0.40, 0.66]
Total (95% CI)		2656		2256	92.7%	0.39 [0.23, 0.67]
Heterogeneity: $Tau^2 = 0.128$; $Chi^2 = 8.81$, df = 1 (P < 0.01); $I^2 = 89\%$						
Test for overall effect: Z = -3.47 (P < 0.01)						
design = RCT						
Triple AXEL 2017	0	95	0	88	0.0%	
AREST 2021	2	41	4	47	7.3%	0.57 [0.11, 2.97]
Test for overall effect: Z = -0.66 (P = 0.51)						
**Total (95% CI)**		**2792**		**2391**	**100.0%**	**0.40 [0.25, 0.65]**
Heterogeneity: $Tau^2 = 0.111$; $Chi^2 = 9.01$, df = 2 (P = 0.01); $I^2 = 78\%$						
Test for overall effect: Z = -3.72 (P < 0.01)						
Test for subgroup differences: $Chi^2 = 0.18$, df = 1 (P = 0.67)						

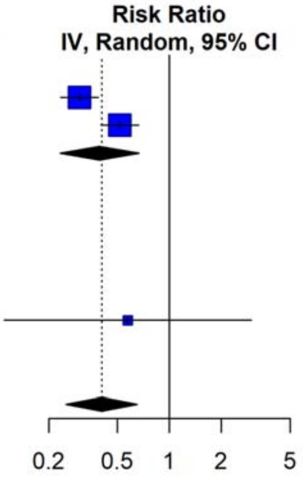

References used in the Supplement.

1. Altavilla R, Caso V, Bandini F, et al. Anticoagulation after Stroke in Patients with Atrial Fibrillation. *Stroke.* 2019;50(8):2093-2100.
2. Butcher KS, Ng K, Sheridan P, et al. Dabigatran Treatment of Acute Noncardioembolic Ischemic Stroke. *Stroke.* 2020;51(4):1190-1198.
3. Cappellari M, Carletti M, Danese A, Bovi P. Early Introduction of Direct Oral Anticoagulants in Cardioembolic Stroke Patients with Non-Valvular Atrial Fibrillation. *Journal of thrombosis and thrombolysis.* 2016;42(3):393-398.
4. De Marchis GM, Seiffge DJ, Schaedelin S, et al. Early Versus Late Start of Direct Oral Anticoagulants after Acute Ischaemic Stroke Linked to Atrial Fibrillation: An Observational Study and Individual Patient Data Pooled Analysis. *Journal of neurology, neurosurgery, and psychiatry.* 2022;93(2):119-125.
5. Paciaroni M, Agnelli G, Falocci N, et al. Early Recurrence and Major Bleeding in Patients with Acute Ischemic Stroke and Atrial Fibrillation Treated with Non-Vitamin-K Oral Anticoagulants (Raf-Noacs) Study. *Journal of the American Heart Association.* 2017;6(12).
6. Seiffge DJ, Traenka C, Polymeris A, et al. Early Start of Doac after Ischemic Stroke: Risk of Intracranial Hemorrhage and Recurrent Events. *Neurology.* 2016;87(18):1856-1862.
7. Toyoda K, Arihiro S, Todo K, et al. Trends in Oral Anticoagulant Choice for Acute Stroke Patients with Nonvalvular Atrial Fibrillation in Japan: The Samurai-Nvaf Study. *International journal of stroke : official journal of the International Stroke Society.* 2015;10(6):836-842.
8. Tsivgoulis G, Katsanos AH, Seiffge DJ, et al. Fatal Intracranial Haemorrhage Occurring after Oral Anticoagulant Treatment Initiation for Secondary Stroke Prevention in Patients with Atrial Fibrillation. *European journal of neurology.* 2020;27(8):1612-1617.
9. Wilson D, Ambler G, Banerjee G, et al. Early Versus Late Anticoagulation for Ischaemic Stroke Associated with Atrial Fibrillation: Multicentre Cohort Study. *Journal of neurology, neurosurgery, and psychiatry.* 2019;90(3):320-325.
10. Yaghi S, Trivedi T, Henninger N, et al. Anticoagulation Timing in Cardioembolic Stroke and Recurrent Event Risk. *Annals of neurology.* 2020;88(4):807-816.
11. Yaghi S, Mistry E, Liberman AL, et al. Anticoagulation Type and Early Recurrence in Cardioembolic Stroke: The Iac Study. *Stroke.* 2020;51(9):2724-2732.
12. Sterne J, Hernán M, McAleenan A, Reeves B, Higgins J. Chapter 25: Assessing Risk of Bias in a Non-Randomized Study. In: *Higgins Jpt, Thomas J, Chandler J, Cumpston M, Li T, Page Mj, Welch Va (Editors). Cochrane Handbook for Systematic Reviews of Interventions Version 6.3 (Updated February 2022). Available from Www.Training.Cochrane.Org/Handbook.*
13. Al Bakr AI, AlOmar RS, Nada MAF, et al. Timing to Start Anticoagulants after Acute Ischemic Stroke with Non-Valvular Atrial Fibrillation. *Journal of the neurological sciences.* 2020;409:116582.
14. Alrohimi A, Buck B, Jickling G, Shuaib A, Thirunavukkarasu S, Butcher KS. Early Apixaban Therapy after Ischemic Stroke in Patients with Atrial Fibrillation. *Journal of neurology.* 2021;268(5):1837-1846.
15. Alrohimi A, Ng K, Dowlatshahi D, et al. Early Dabigatran Treatment after Transient Ischemic Attack and Minor Ischemic Stroke Does Not Result in Hemorrhagic Transformation. *The Canadian journal of neurological sciences Le journal canadien des sciences neurologiques.* 2020;47(5):604-611.
16. Gioia LC, Kate M, Sivakumar L, et al. Early Rivaroxaban Use after Cardioembolic Stroke May Not Result in Hemorrhagic Transformation: A Prospective Magnetic Resonance Imaging Study. *Stroke.* 2016;47(7):1917-1919.

17. Frisullo G, Profice P, Brunetti V, et al. Prospective Observational Study of Safety of Early Treatment with Edoxaban in Patients with Ischemic Stroke and Atrial Fibrillation (Sates Study). *Brain Sci.* 2020;11(1):30.
18. Seiffge DJ, Paciaroni M, Wilson D, et al. Direct Oral Anticoagulants Versus Vitamin K Antagonists after Recent Ischemic Stroke in Patients with Atrial Fibrillation. *Annals of neurology.* 2019;85(6):823-834.
